# Monoclonal antibodies targeting nonstructural viral antigens can activate ADCC against human cytomegalovirus

**DOI:** 10.1172/JCI139296

**Published:** 2021-02-15

**Authors:** Virginia-Maria Vlahava, Isa Murrell, Lihui Zhuang, Rebecca J. Aicheler, Eleanor Lim, Kelly L. Miners, Kristin Ladell, Nicolás M. Suárez, David A. Price, Andrew J. Davison, Gavin W.G. Wilkinson, Mark R. Wills, Michael P. Weekes, Eddie C.Y. Wang, Richard J. Stanton

**Affiliations:** 1 Division of Infection and Immunology, School of Medicine, Cardiff University, Cardiff, United Kingdom.; 2 Cardiff Metropolitan University, Cardiff, United Kingdom.; 3 Department of Medicine, University of Cambridge, Cambridge, United Kingdom.; 4 University of Glasgow-MRC Centre for Virus Research, Glasgow, United Kingdom.; 5 Cambridge Institute for Medical Research, University of Cambridge, Cambridge, United Kingdom.

**Keywords:** Immunology, Virology, NK cells, Proteomics

## Abstract

Human cytomegalovirus (HCMV) is a ubiquitous pathogen that causes severe disease following congenital infection and in immunocompromised individuals. No vaccines are licensed, and there are limited treatment options. We now show that the addition of anti-HCMV antibodies (Abs) can activate NK cells prior to the production of new virions, through Ab-dependent cellular cytotoxicity (ADCC), overcoming viral immune evasins. Quantitative proteomics defined the most abundant HCMV proteins on the cell surface, and we screened these targets to identify the viral antigens responsible for activating ADCC. Surprisingly, these were not structural glycoproteins; instead, the immune evasins US28, RL11, UL5, UL141, and UL16 each individually primed ADCC. We isolated human monoclonal Abs (mAbs) specific for UL16 or UL141 from a seropositive donor and optimized them for ADCC. Cloned Abs targeting a single antigen (UL141) were sufficient to mediate ADCC against HCMV-infected cells, even at low concentrations. Collectively, these findings validated an unbiased methodological approach to the identification of immunodominant viral antigens, providing a pathway toward an immunotherapeutic strategy against HCMV and potentially other pathogens.

## Graphical abstract

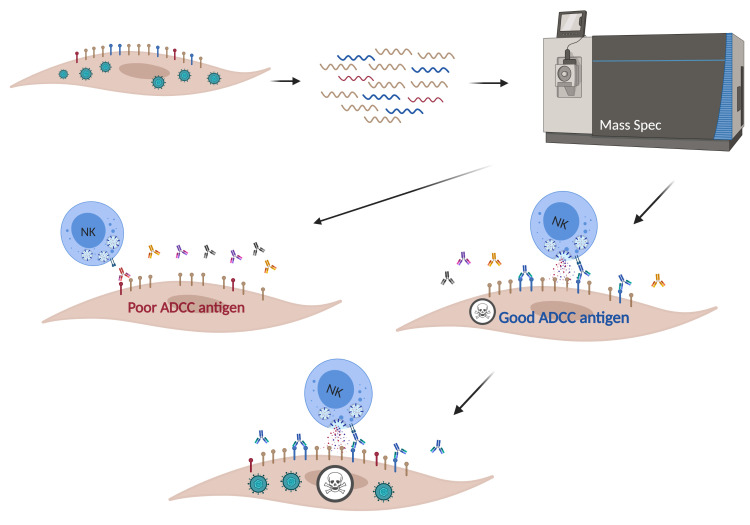

## Introduction

Human cytomegalovirus (HCMV) establishes lifelong infection in the face of robust humoral and cell-mediated immune responses. The virus is a significant cause of morbidity and mortality in immunocompromised individuals such as transplant recipients and patients with HIV and following congenital infection. A vaccine against HCMV is considered to be the highest priority, particularly for the prevention of congenital disease (1), but none has been licensed. The standard for treatment is therefore antiviral agents, however, these are limited by toxicity and the emergence of resistant strains (2).

As an alternative, antibody (Ab) responses have been investigated as a basis for improved vaccines and immunotherapies (3–9). Several lines of evidence support a protective role for Abs in infection, including observational studies of natural immunity, which have documented a correlation between Ab titers and the prevention of intrauterine transmission (10–13). Moreover, the administration of hyperimmune globulin (HIG) can improve survival in patients undergoing solid organ transplantation (14), and Ab titers correlated with protection in vaccine trials (8, 9). As a result, a range of Abs directed against virion envelope glycoproteins that are capable of neutralizing the entry of cell-free virus have been developed (5, 15, 16). However, neutralizing monoclonal antibodies (mAbs) administered as therapies have had only modest effects and/or failed to meet primary endpoints in clinical trials, namely, a reduction in viremia and/or the need for preemptive therapy (17, 18).

One potential explanation for this lack of clinical efficacy lies in the biology of virus dissemination. Spread between individuals involves cell-free virus, which can be efficiently inhibited by neutralizing Abs. In contrast, dissemination within a host likely relies primarily on direct cell-to-cell spread (19–24), which is resistant to neutralizing antibodies (25), irrespective of the Ab repertoire of the donor (26). Thus, although classical neutralizing Abs may have a role in preventing transmission between people, they may be less effective in preventing the spread of virus within an individual. This is consistent with clinical trials of a subunit gB vaccine, in which protection correlated with Ab levels, but the induced antibodies did not exhibit overt neutralizing activity (27, 28). We therefore sought to prioritize Ab-based immunotherapeutic approaches that could target infected cells directly.

NK cells are crucial for virus control in vivo (29). This fact is highlighted by the impressive arsenal of HCMV-encoded immune evasins that act in consort to suppress NK cell activation through the manipulation of ligands for activating and inhibitory receptors (30, 31). However, in addition to working through these receptors, NK cells participate in Ab-dependent cellular cytotoxicity (ADCC) (32, 33). ADCC involves the activation of NK cells upon engagement of Fc receptors (FcRs) on the NK cell surface, with the Fc portion of an Ab bound to a target cell. In vivo, HCMV infection is associated with a dramatic expansion of “adaptive” NK cells marked by the expression of CD94/NKG2C, and CD57 and by the loss of FcεR1γ (29, 34). These cells are exceptionally efficient at mediating ADCC (35–38) and have been associated with protection from disease (35, 39–41). Accordingly, ADCC may be an important mechanism of immune control during natural infection. In this scenario, Abs act as critical stimulators of cellular immunity, rather than acting through virus neutralization.

We were therefore interested in how ADCC operated in the context of an HCMV infection and whether it could be exploited for therapeutic use. We found that anti-HCMV Abs could activate NK cells early after HCMV infection, prior to the production of new virions, and that these Abs had a remarkable capacity to overwhelm the potent HCMV-encoded NK cell evasion mechanisms in vitro. We have previously exploited the power of proteomics to characterize viral and host gene expression during HCMV infection in unparalleled detail, revealing the ways in which the virus manipulates the host cell to promote survival, and to identify ways of counteracting the virus through antiviral restriction factors (33, 42–47). Here, we combined this technique with functional immunological screening to identify the targets on the infected cell surface that mediate antiviral ADCC. Surprisingly, these techniques revealed that the optimal targets were not the structural glycoproteins that are traditionally assumed to be ADCC targets, but immune evasins that are expressed earlier during the viral life cycle. Their identification enabled us to isolate human mAbs directed against these targets that, once we had genetically engineered them, could activate NK cells in response to HCMV-infected cells. Thus, our technologies enabled the identification of optimal antigenic targets for the development of antiviral therapeutics, and the isolation of what we believe to be the first human mAbs targeting a single HCMV protein that are sufficient to mediate enhanced NK activation through ADCC, despite virus-encoded immune evasins. Our platform is therefore capable of generating novel antiviral immunotherapies that can efficiently activate antiviral cellular immunity.

## Results

### HCMV-infected cells are susceptible to ADCC during the early phase of infection.

We examined the ability of Cytotect (clinical-grade HIG pooled from donors with high anti-HCMV–neutralizing titers) to enhance NK cell activation in the presence of target cells infected with a HCMV strain (Merlin) expressing the complete repertoire of virally encoded immune evasins. Since adaptive NK cells are the primary mediators of ADCC in PBMCs from HCMV-seropositive donors (29, 35–38), we examined the activation of CD56^+^ NK cells in the CD57^+^ and NKG2C^+^ subsets, measuring degranulation via surface mobilization of CD107a. Both cell populations demonstrated a greater enhancement of degranulation when Ab was added, compared with the NKG2C^–^CD57^–^ cell population. However, in the majority of donors, we observed a large overlap between the CD57^+^ and NKG2C^+^ cell populations, and the levels of degranulation were virtually indistinguishable between them. As NKG2C^+^ NK cells are rarely present in uninfected individuals, and up to 4% of people do not harbor the corresponding gene (KLRC2), subsequent data were recorded for CD57^+^ NK cells.

**Figure 1:**
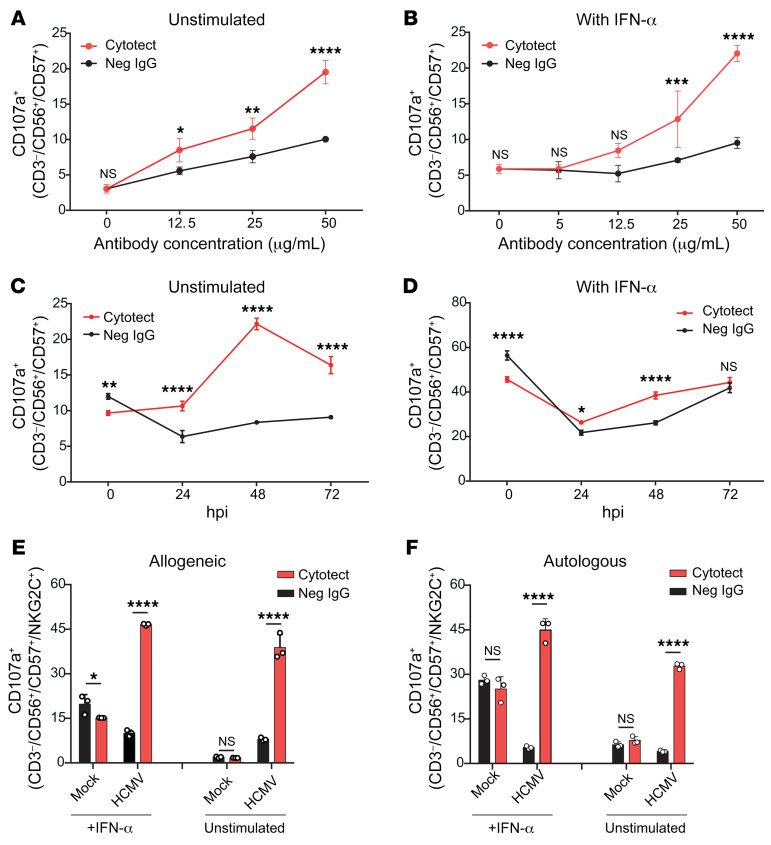
Characterization of ADCC-mediated NK cell activation against HCMV-infected fibroblasts. HFFFs immortalized with hTERT or similarly immortalized autologous SFs were infected with HCMV strain Merlin. Mock-infected HF-TERTs or SFs were included as controls. (**A** and **B**) Percentage of degranulation of CD56^+^CD57^+^ NK cells among PBMCs in the presence of HF-TERTs infected for 48 hours with HCMV and different concentrations of either Cytotect or seronegative IgGs (Neg IgG). PBMCs were either untreated (**A**) or pretreated for 18 hours with IFN-α (**B**). (**C** and **D**) Percentage of degranulation of CD56^+^CD57^+^ NK cells among PBMCs in the presence of HF-TERTs infected for 24 hours, 48 hours, or 72 hours with HCMV and either Cytotect or seronegative IgGs (each at 50 μg/mL). PBMCs were either untreated (**C**) or pretreated for 18 hours with IFN-α (**D**). (**E** and **F**) Percentage of degranulation of CD56^+^CD57^+^NKG2C^+^ NK cells among PBMCs in the presence of HF-TERTs (**E**) or SFs (**F**) infected for 48 hours with HCMV and either Cytotect or seronegative IgGs (each at 50 μg/mL). Results are representative of at least 3 experiments. All data are shown as the mean ± SD of triplicate samples. *P < 0.05, **P < 0.01, ***P < 0.001, and ****P < 0.0001, by 2-way ANOVA.

Cytotect enhanced NK cell activation at a minimum concentration of 12.5 μg/mL and became progressively more potent as concentrations increased to 50 μg/mL, representing a relatively steep activation curve (Figure 1A). Experiments were capped at this maximum, because increased background activation was observed with higher concentrations of IgG Abs from HCMV-seronegative donors. Interestingly, efficacy was not dependent on NK cell stimulation, since equivalent results were obtained whether or not cells were preincubated with IFN-α (Figure 1, A and B). Given that HCMV actively represses the release of IFNs (48), this supports an important role for ADCC in rapidly activating NK cells against HCMV without a requirement for additional stimulations.

When the sensitivity of HCMV-infected cells to ADCC was investigated over the course of infection, we detected NK cell activation as early as 24 hours post infection (hpi), irrespective of preincubation with IFN-α, but this increased dramatically at 48 hpi (Figure 1, C and D) before decreasing slightly at 72 hpi. This reduction may be related to the expression at this later time point of viral FcRs and other NK inhibitors, which antagonize ADCC (32, 45, 49). HCMV antigens expressed on the cell surface by 48 hpi are therefore recognized by naturally occurring Abs and act as effective targets to drive ADCC. Importantly, HCMV has a slow replication cycle, with virions not produced in significant numbers until 72 hpi, so these observations highlighted a therapeutic opportunity to limit the dissemination of HCMV.

HCMV downregulates, but does not abrogate, the expression of endogenous HLA class I molecules. NK cell activation may therefore be influenced by interactions between residual HLA-I and killer immunoglobulin-like receptors (KIRs). To address this possibility, we investigated NK cell recognition of allogeneic and autologous targets in the context of ADCC. The potency of HCMV-encoded NK cell evasion functions is illustrated by the fact that uninfected autologous and allogeneic targets activated NK cells much more efficiently than did the corresponding HCMV-infected targets (Figure 1, E and F). However, in both cases, the inclusion of seropositive Abs overcame the strong protective effects of HCMV-encoded NK evasion functions to stimulate high levels of NK cell activation, irrespective of preincubation with IFN-α (Figure 1, E and F). Thus, the addition of anti-HCMV Abs was able to potently activate NK cells and overcome viral immune evasion prior to the production of new virions, irrespective of NK cell stimulation or engagement of HLA-I.

### Antigens expressed on the cell surface at 48 hpi promote ADCC.

**Figure 2:**
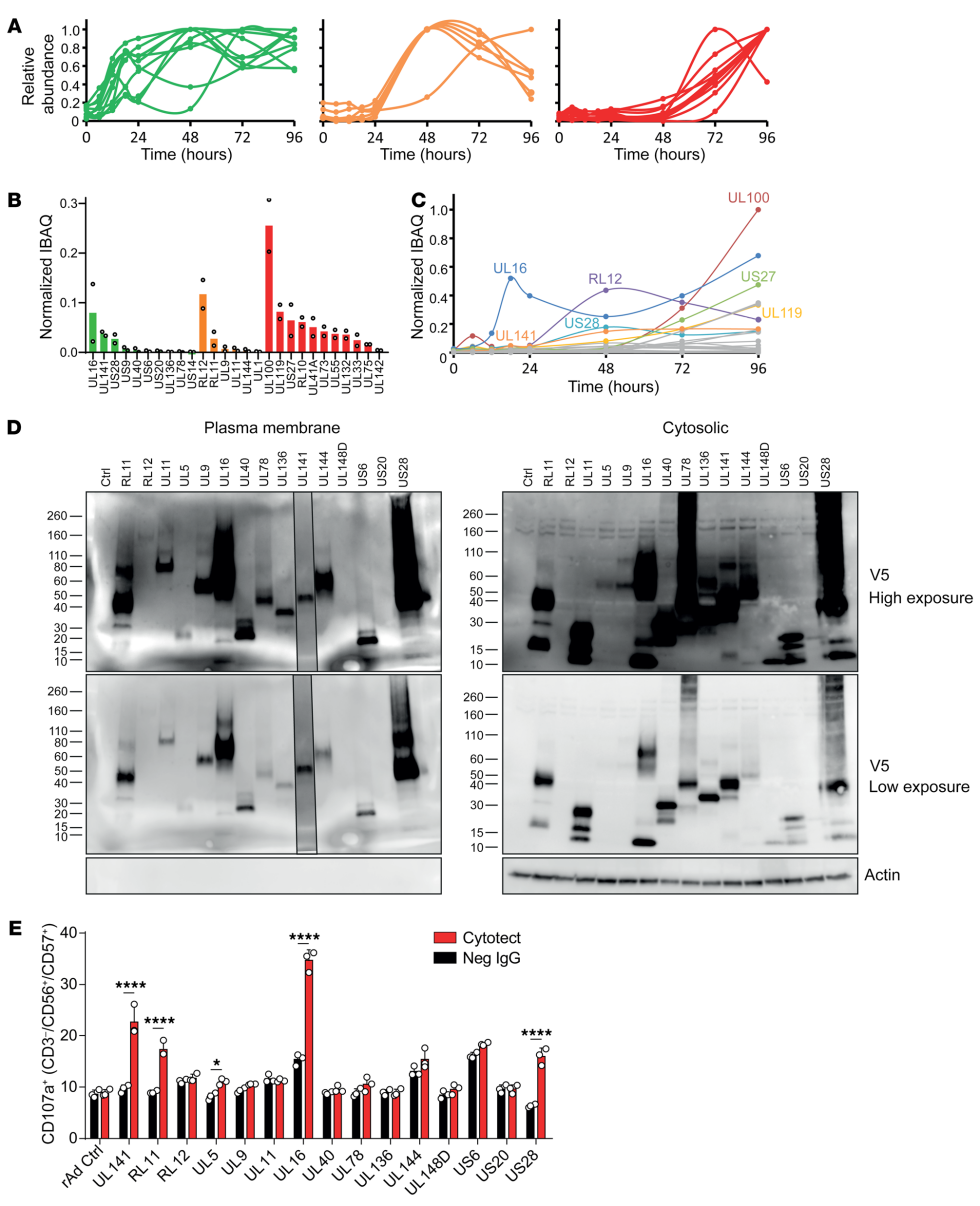
Identification of viral proteins on the plasma membrane that could prime ADCC. (**A**) Temporal profiles of viral proteins (*n* = 27) identified previously on the surface of cells infected with HCMV. Proteins were only included in the analysis if detected in experiments PM1 and PM2 and quantified by 2 or more peptides in experiment PM1 or experiment PM2. Data are shown for experiment PM2. Proteins are grouped on the basis of expression kinetics, indicating that greater than 25% of the maximal signal was reached by 24 hours (left), 48 hours (middle), or 72 hours (right). (**B**) Average total abundance of each surface-expressed viral protein measured using IBAQ. Error bars indicate ranges from experiments PM1 and PM2. (**C**) Partitioned IBAQ abundance of each surface-expressed viral protein over time. Average IBAQ abundance values in **B** were multiplied by the fractional abundance at each time point from **A**. (**D**) HF-TERTs transfected with the coxsackie-adenovirus receptor (HFFF-hCARs) were transduced with RAds expressing individual viral proteins. An identical vector lacking a transgene was used as a control. Surface-expressed proteins were isolated by aminooxy biotinylation followed by immunoprecipitation with streptavidin beads 48 hours after transduction. Western blots show detection of the C-terminal V5 tags engineered into each protein, with the exception of UL141, which was detected with a UL141-specific Ab. UL141 staining of the gel was performed separately but is overlaid on the same image. (**E**) Percentage of degranulation of CD56^+^CD57^+^ NK cells among PBMCs in the presence of HFFF-hCARs, transduced as in **D**, and either Cytotect or seronegative IgGs (each at 50 μg/mL). Results are representative of 3 experiments. Data are shown as the mean ± SD of triplicate samples (**E**). *P < 0.05 and ****P < 0.0001, by 2-way ANOVA. ctrl, control.

ADCC has the potential to target infected cells during the early phase of the HCMV replication cycle. To determine which viral antigens primed ADCC, we reanalyzed data from our quantitative temporal viromics investigation of the HCMV-infected cell-surface proteome (45). We identified 3 clear kinetic classes of protein expression (Figure 2A). Ten proteins reached at least 25% of their maximal cell-surface levels by 24 hpi, and an additional 5 proteins reached at least 25% of their maximal levels by 48 hpi. Thus, a substantial number of viral proteins are trafficked to the cell membrane prior to the production of new virions. Furthermore, multiple proteins reached a maximal overall abundance equal to or higher than that of structural proteins expressed during the later phases of infection (Figure 2B). Therefore, targeting proteins expressed early during the viral life cycle is likely to be equally as effective as targeting later-expressed factors. An analysis of the partitioned abundance of each protein over time indicated that UL16, RL12, UL141, and US28 were expressed on the cell surface at 48 hpi, were among the most abundant viral proteins at this time point, and would therefore be potential candidates for ADCC targets (Figure 2C).

On the basis of these results, we generated replication-deficient adenovirus (RAd) vectors expressing each of the 15 viral proteins that were reproducibly identified on the surface of HCMV-infected cells by 48 hpi (Figure 2D). Each RAd was then tested individually for its capacity to promote ADCC in the presence of pooled polyclonal HIG (Figure 2E). UL16, UL141, US28, RL11, and UL5 each induced a significant increase in NK cell activation that was dependent on the presence of Cytotect, indicating that these viral antigens could induce early-phase ADCC.

### Abs directing ADCC can be isolated from human donors.

**Figure 3:**
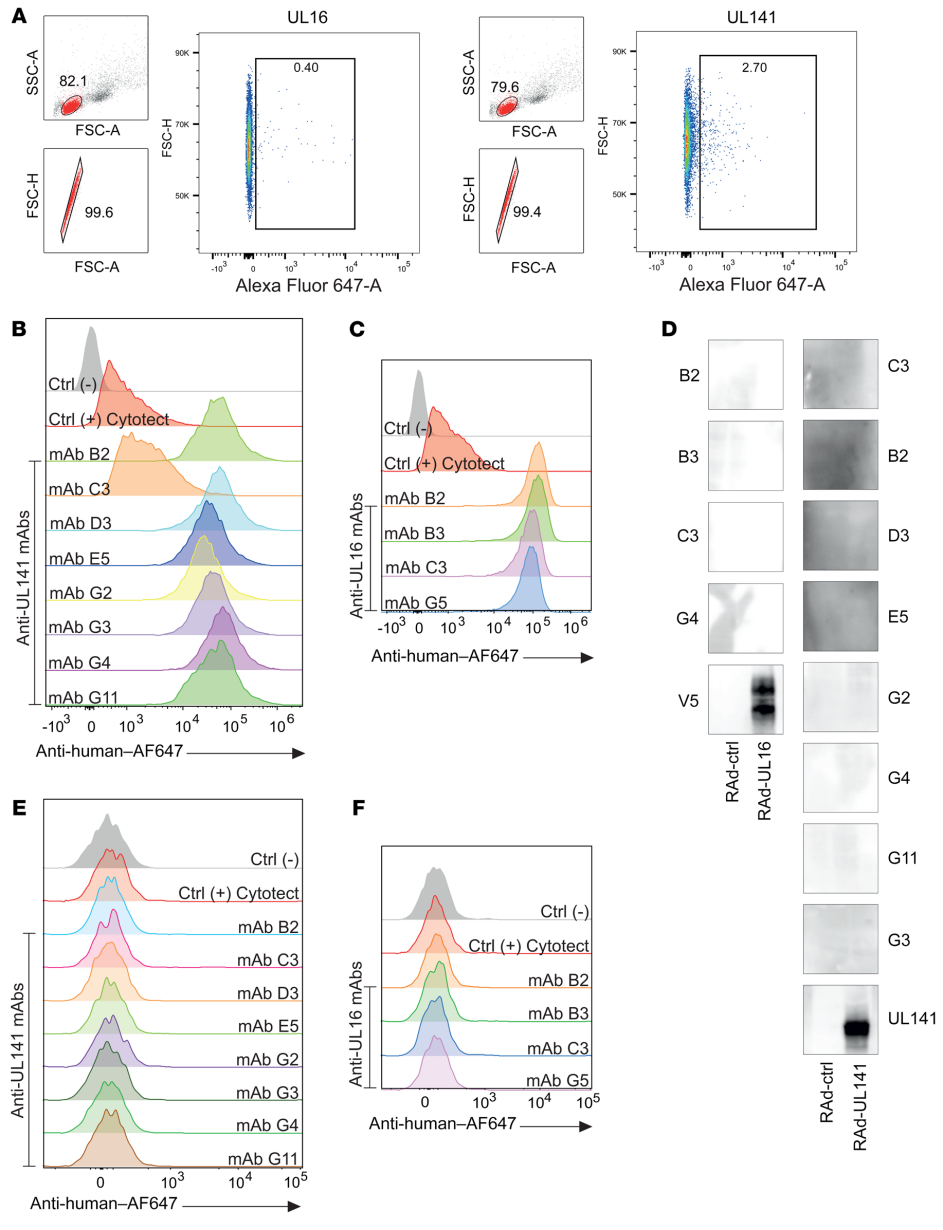
Anti-UL16 and anti-UL141 mAbs can be isolated and cloned from seropositive donors. (**A**) IgG^+^ B cells from a HCMV-seropositive donor were stained with fluorescently labeled UL16 or UL141 proteins to sort B cells expressing specific mAbs. FSC, forward scatter; SSC, side scatter. (**B** and **C**) HFFF-hCARs were transduced with RAds expressing UL141 or UL16 lacking their ER retention signals. Cells were stained with the cloned human anti-UL141 or anti-UL16 mAbs and analyzed by flow cytometry. Cytotect was used as a positive control. (**D**) HFFF-hCARs were transduced with RAds lacking a transgene, or RAds expressing wild-type forms of UL141 or UL16. Samples were lysed, separated by SDS-PAGE, and analyzed by immunoblotting using human anti-UL16 or anti-UL141 mAbs. As a positive control, the UL16 lysate was stained with an anti-V5 Ab, and the UL141 lysate was stained with a murine anti-UL141 Ab. (**E** and **F**) HFFF-hCARs were transduced with RAds expressing wild-type forms of UL141 or UL16. Forty-eight hours later, they were stained with human anti-UL141 or anti-UL16 mAbs or Cytotect and then analyzed by flow cytometry.

To investigate whether the identified viral protein targets could mediate ADCC in the context of HCMV infection, we generated a series of mAbs. RL11 is an Fc-binding protein (50) that complicates both the production of specific Abs and the analysis of functional assays. US28 is a type 3 transmembrane protein, and thus the generation of US28-specific Abs would be less straightforward. Therefore, RL11 and US28 may not provide routine target antigens. Further, since UL5 was associated with only modest levels of NK cell activation, the type 1 membrane proteins UL16 and UL141 were prioritized. Sequences encoding the extracellular domains of each protein were cloned as modified constructs with a C-terminal 6xHis-tag (UL16) or a C-terminal Strep-tag (UL141) into separate RAd vectors for expression. The corresponding proteins were purified from cell supernatants via affinity chromatography, labeled with fluorochromes, and used as probes to stain IgG^+^ B cells from a donor infected with HCMV. UL141-specific B cells were more numerous than UL16-specific B cells (Figure 3A). Single antigen-specific B cells were then flow-sorted into culture medium containing CD40L^+^ feeders, IL-2, IL-4, IL-21, and B cell activating factor (BAFF) to generate plasma cells (51). All secreted mAbs were then screened against cells expressing UL16 or UL141. Both proteins contain an ER retention signal in the C-terminal cytoplasmic domain, which restricted cell-surface expression (Supplemental Figure 1A; supplemental material available online with this article; https://doi.org/10.1172/JCI139296DS1). To increase the sensitivity of this flow cytometry–based Ab screen, we increased the cell-surface abundance of target antigens by deleting this region (Supplemental Figure 1A). Screening 60 B cell supernatants against these proteins revealed that 9 bound UL141 and 5 bound UL16 (Supplemental Figure 1B).

B cell receptor (BCR) sequencing revealed that the predicted amino acid sequences of these mAbs were diverse and incorporated both κ and λ light chains, suggesting that Abs had the potential to target distinct epitopes (Supplemental Figure 2). We subcloned the variable domains of these BCRs into an expression plasmid that provided a human IgG1 backbone, with the specific purpose of optimizing the utility of the Ab fusion for ADCC. When expressed, these recombinant human mAbs retained their capacity to bind to UL141 and UL16 on the cell surface (Figure 3, B and C), but not to denatured antigen (Figure 3D), suggesting that all bind to conformational epitopes.

### Anti-UL16 and anti-UL141 human mAbs activate ADCC when antigen is expressed in isolation.

**Figure 4:**
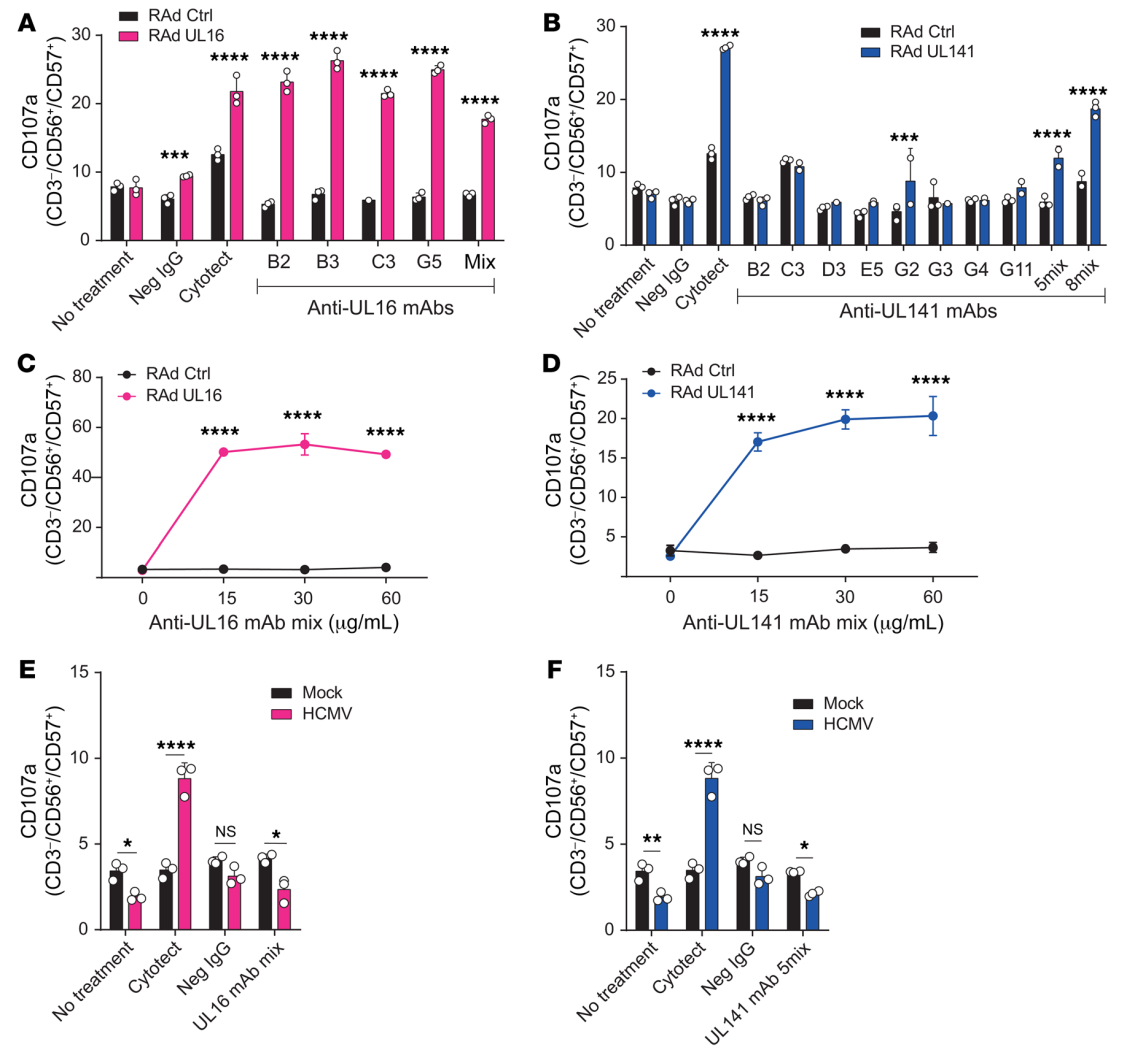
Human anti-UL16 and anti-UL141 mAbs activate ADCC efficiently against adenovirally expressed UL16 and UL141. (**A**–**D**) HFFF-hCARs were transduced with RAds expressing wild-type UL16 or UL141. An identical vector lacking a transgene was used as a control. (**A**) Percentage of degranulation of CD56^+^CD57^+^ NK cells among PBMCs in the presence of transduced HFFF-hCARs and Cytotect (40 μg/mL), seronegative IgGs (40 μg/mL), or UL16-specific mAbs (each at 30 μg/mL). All 4 mAbs were included at equimolar concentrations in the mixture. (**B**) As in **A** for UL141. Five mAbs were included at equimolar concentrations in 1 mixture (B2, D3, G3, G4, and G11), and 8 mAbs were included at equimolar concentrations in another mixture (B2, C3, D3, E5, G2, G3, G4, and G11). (**C**) Percentage of degranulation of CD56^+^CD57^+^ NK cells among PBMCs in the presence of transduced HFFF-hCARs and different concentrations of the tetravalent UL16-specific mAb mixture. (**D**) As in **C** for the pentavalent UL141-specific mAb mixture. (**E** and **F**) HF-TERTs were infected with HCMV strain Merlin. Mock-infected HF-TERTs were included as controls. (**E**) Percentage of degranulation of CD56^+^CD57^+^ NK cells among PBMCs in the presence of infected HF-TERTs and Cytotect, seronegative IgGs, or the UL16-specific mAb mixture (each at 30 μg/mL). (**F**) As in **E** for UL141. Results are representative of at least 3 experiments. Data are shown as the mean ± SD of triplicate samples (**A**–**F**). All experiments were performed 48 hours after transduction (**A**–**D**) or infection (**E** and **F**). *P < 0.05, **P < 0.01, ***P < 0.001, and ****P < 0.0001, by 2-way ANOVA.

Although the mAbs bound to UL16 and UL141 when optimized for high expression on the cell surface (Figure 3, B and C), binding to the natural forms was not detectable by flow cytometry (Figure 3, E and F, Supplemental Figure 1A), indicating that very low levels of these proteins naturally traffic to the cell surface. Nevertheless, ADCC assays appeared more sensitive than flow cytometry, as the natural versions of both genes were able to induce ADCC with both Cytotect and mAbs (Figure 4, A and B).

Each novel UL16 mAb was readily able to drive ADCC against fibroblasts expressing wild-type UL16 with an efficiency comparable to that observed with Cytotect (Figure 4A). The level of ADCC elicited by different anti-UL16 mAbs was remarkably similar, despite the diversity of their antigen binding (Fab) sequences. When the 5 mAbs were mixed together at equimolar concentrations, the ADCC effect was not enhanced beyond the level of each individual Ab. These findings suggested that each mAb targeted the same immunodominant epitope with similar efficiency, irrespective of diversity in the corresponding antigen-binding domains.

In contrast, only 2 of the UL141-specific mAbs were capable of mediating ADCC in isolation, and activation was extremely weak (Figure 4B). However, when all 8 purified Abs were mixed together at equal concentrations, ADCC was efficiently activated. Three of the Abs were prone to eliciting nonspecific activation against control infected cells, and therefore we tested a mixture of the other 5 Abs and found them to be equally capable of activating ADCC, but with reduced background levels (Figure 4B). The fact that anti-UL141 mAbs stimulated higher levels of degranulation when used as a mixture suggests that at least some of them bind to different epitopes on UL141. In dose-titration experiments against the corresponding targets, mixtures of UL16-specific or UL141-specific mAbs maximally activated NK cells at concentrations above 15 μg/mL (Figure 4, C and D), indicating greater efficacy compared with Cytotect (Figure 1, A and B).

Although these results were encouraging in terms of therapeutic development, pooled mAbs specific for UL16 or UL141 were unable to activate NK cells in the presence of targets infected with HCMV, even though Cytotect was effective (Figure 4, E and F). HCMV encodes 4 Fc-binding proteins (FcRs) (RL11, RL12, RL13, and UL119) that have the potential to antagonize ADCC. Accordingly, human IgGs bound cells infected with an HCMV-mutant strain lacking all 4 of these genes (HCMVΔFc) to a lesser extent than they bound cells infected with wild-type HCMV (Supplemental Figure 3A). However, NK cells were activated similarly under both conditions in the presence of Cytotect (Supplemental Figure 3B). The lack of efficacy of the specific Abs against HCMV-infected cells was therefore not caused by antagonism of ADCC by viral FcRs. It may reflect lower levels of protein on the cell surface during HCMV infection compared with RAd expression (Supplemental Figure 3C), or the concerted action of multiple virally encoded immune evasins that inhibit NK activation (30).

### Ab engineering enables mAbs to activate ADCC against HCMV.

**Figure 5:**
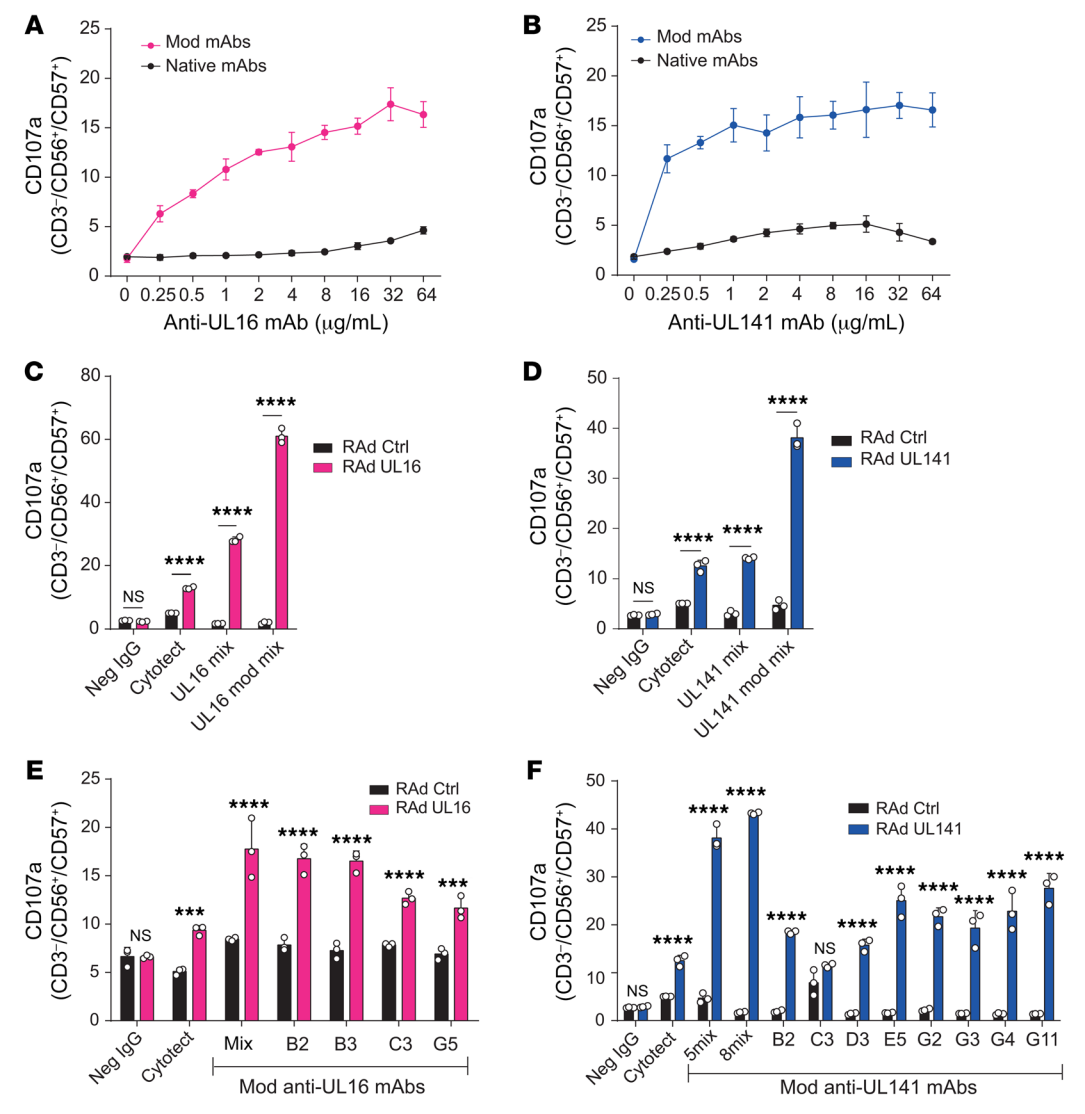
Optimized anti-UL16 and anti-UL141 mAbs activate ADCC efficiently against adenovirally expressed UL16 and UL141. HFFF-hCARs were transduced with RAds expressing wild-type UL16 or UL141. An identical vector lacking a transgene was used as a control. (**A**) Percentage of degranulation of CD56^+^CD57^+^ NK cells among PBMCs in the presence of transduced HFFF-hCARs and different concentrations of native or Fc-engineered (modified) UL16-specific mAbs (tetravalent mixes). (**B**) As in **A** for UL141 (pentavalent mixes). (**C**) Percentage of degranulation of CD56^+^CD57^+^ NK cells among PBMCs in the presence of transduced HFFF-hCARs and Cytotect, seronegative IgGs, or tetravalent mixes of native or Fc-engineered (modified) UL16-specific mAbs (native Abs each at 30 μg/mL; Fc-engineered [modified] mAbs each at 1 μg/mL). (**D**) As in **C** for UL141 (pentavalent mixes). (**E**) As in **C** for individual Fc-engineered (modified) UL16-specific mAbs. (**F**) As in **D** for individual Fc-engineered (modified) UL141-specific mAbs. Results are representative of at least 3 experiments. All data are shown as the mean ± SD of triplicate samples. All experiments were performed 48 hours after transduction. ***P < 0.001 and ****P < 0.0001, by 2-way ANOVA. Mod, modified.

A major advantage of cloned mAbs is that they can be manipulated to enhance different effector functions. We took advantage of this to optimize the ability of our mAbs to activate ADCC by introducing 2 amino acid sequence changes into the Fc region that had previously been shown to enhance binding to CD16 on NK cells (52). In line with previous data indicating that viral and host FcRs bind Fc in different ways (53), these modifications did not affect binding to viral FcRs (Supplemental Figure 3, D and E). Dose-titration experiments revealed that mixtures of engineered mAbs specific for UL16 or UL141 activated NK cells more potently and at much lower concentrations than did the corresponding unmodified mAbs (Figure 5, A and B) or Cytotect (Figure 5, C and D). As before, when tested separately, all of the mAbs against UL16 activated ADCC, and we observed no increase in activation when they were combined (Figure 5E). However, unlike the unmodified versions, all the modified UL141 mAbs activated ADCC individually (Figure 5F). Moreover, they retained the ability to show enhanced activation when used in combination, whether as a mixture of 5 or 8 mAbs (Figure 5F).

**Figure 6:**
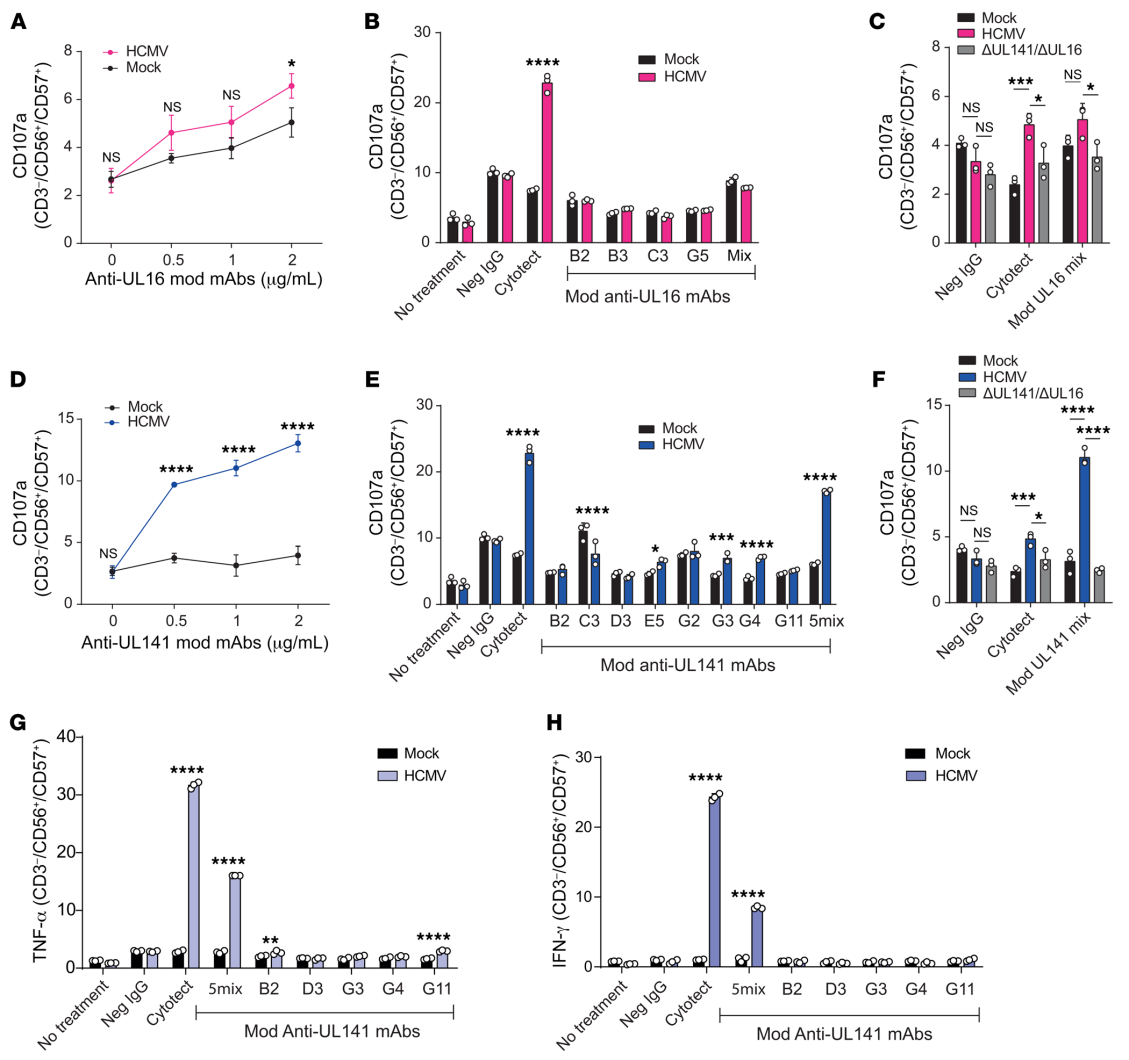
Anti-UL141–optimized Abs activate ADCC efficiently against HCMV. HF-TERTs were infected with HCMV strain Merlin (**A**–**H**) or Merlin ΔUL16 ΔUL141 (**C** and **F**). Mock-infected HF-TERTs were included as controls. (**A**) Percentage of degranulation of CD56^+^CD57^+^ NK cells among PBMCs in the presence of infected HF-TERTs and different concentrations of Fc-engineered (modified) UL16-specific mAbs (tetravalent mixture). (**B**) Percentage of degranulation of CD56^+^CD57^+^ NK cells among PBMCs in the presence of infected HF-TERTs and Cytotect (40 μg/mL), seronegative IgGs (40 μg/mL), or Fc-engineered (modified) UL16-specific mAbs tested individually or in combination (each at 1 μg/mL). (**C**) Percentage of degranulation of CD56^+^CD57^+^ NK cells among PBMCs in the presence of infected HF-TERTs and Cytotect (40 μg/mL), seronegative IgGs (40 μg/mL), or the tetravalent mixture of Fc-engineered (modified) UL16-specific mAbs (each at 1 μg/mL). Activity was tested against HF-TERTs infected with Merlin or Merlin ΔUL16 ΔUL141. (**D**) As in **A** for UL141 (pentavalent mixture). (**E**) As in **B** for UL141. (**F**) As in **C** for UL141. (**G**) Percentage of intracellular TNF-α production by CD56^+^CD57^+^ NK cells among PBMCs in the presence of infected HF-TERTs and Cytotect (50 μg/mL), seronegative IgGs (50 μg/mL), or Fc-engineered (modified) UL141-specific mAbs tested individually or in combination (each at 1 μg/mL). (**H**) As in **G** for IFN-γ. Results are representative of at least 3 experiments. Data are shown as the mean ± SD of triplicate samples (**A**–**H**). Experiments were performed 48 hours after infection (**A**–**F**). *P < 0.05, **P < 0.01, ***P < 0.001, and ****P < 0.0001, by 2-way ANOVA.

Next, we tested the efficiency of the mAbs in the context of HCMV infection both separately and in combination. Even in their modified form, the anti-UL16 mAbs were not able to reproducibly activate ADCC against HCMV-infected cells (Figure 6, A–C). In contrast, ADCC was efficiently achieved against HCMV using the modified anti-UL141 mAbs. Individually, we found that these mAbs only activated ADCC very weakly, but the combination of 5 Abs was successful at activating ADCC almost as effectively as Cytotect, despite being used at a 40-fold lower concentration (Figure 6, D and E). This effect was highly specific, because activation was not apparent when a virus lacking the cognate antigen was used (Figure 6F). Furthermore, these Abs were also capable of activating NK cells to secrete TNF-α and IFN-γ, indicating potent antiviral effector functions in the presence of targets infected with HCMV (Figure 6, G and H).

**Figure 7:**
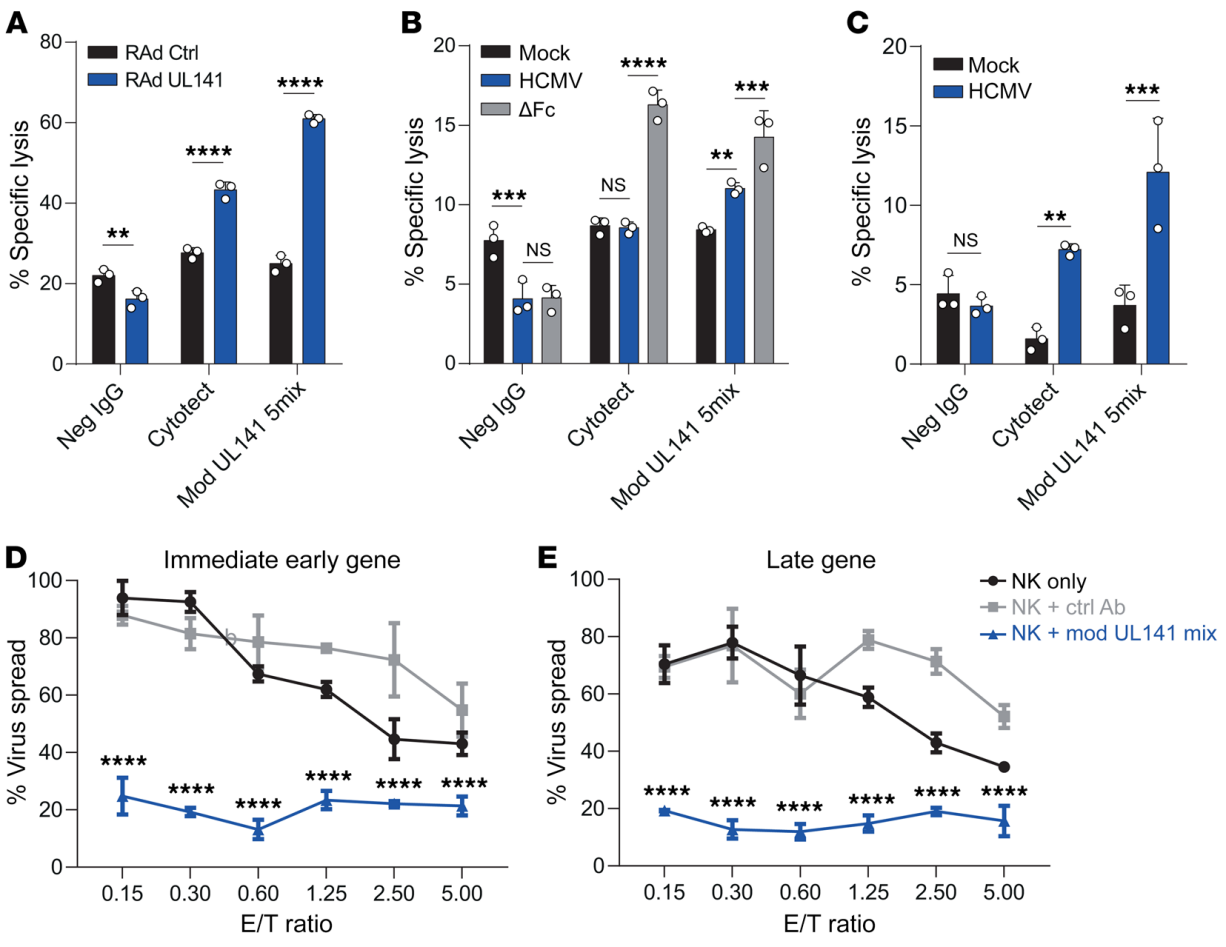
Anti-UL141–optimized Abs mediate efficient killing of HCMV-infected cells. (**A**–**C**) ^51^Cr release into the supernatant was used as a measure of the ability of NK cells to kill target cells. Targets were mixed with ex vivo–purified NK cells as effectors at a an E/T ratio of 20:1, and ^51^Cr release was measured 4 hours later. Seronegative IgG (50 μg/mL), Cytotect (50 μg/mL), or a mixture of 5 Fc-engineered (modified) UL141-specific mAbs were included as indicated. Targets were HF-CARs infected with RAd vectors expressing UL141 (RAd-UL141), or lacking a transgene (RAd Ctrl) (**A**); HFFF mock infected or infected with wild-type HCMV (HCMV) or HCMV lacking the viral FcRs (ΔFc) (**B**); or ARPE19 mock infected or infected with wild-type HCMV (**C**). For ARPE19 infection, cells were infected by coculturing with purified fibroblasts for 24 hours and then sorted to purity. All experiments were performed 48 hours after infection. (**D** and **E**) HCMV expressing mCherry linked to an immediate early gene (UL36), and EGFP linked to a late gene (UL32) were used to infect SFs at a low MOI. Autologous NK cells were then added alone or together with a control mAb or the mixture of 5 modified anti-UL141 mAbs (each at 1 μg/mL). Eight to 10 days later, the percentage of infected cells demonstrating expression of immediate early (**D**) or late (**E**) viral proteins were measured by flow cytometry for mCherry or EGFP, respectively, and normalized to the percentage of infected cells in the absence of NK cells. Results are representative of at least 2 experiments. Data are shown as the mean ± SD of triplicate samples. **P < 0.01, ***P < 0.001, and ****P < 0.0001, by 2-way ANOVA.

Finally, we examined the ability of our mAbs to promote direct killing of cells. Measuring short-term cytotoxicity using chromium-release assays revealed that a mixture of 5 modified anti-UL141 Abs led to a substantial increase in NK-mediated cell death when UL141 was expressed in isolation (Figure 7A), or when fibroblasts were infected with HCMV (Figure 7B). This effect was not restricted by cell type, because we obtained similar results when HCMV infected epithelial cells were used (Figure 7C). Furthermore, our defined Abs markedly outperformed Cytotect in these assays, despite being used at a lower concentration. Interestingly, unlike in degranulation assays (Supplemental Figure 3B), when we performed cytotoxicity experiments, the viral FcRs did limit cell death, since killing was significantly enhanced in their absence (Figure 7B). However, this effect was more pronounced with Cytotect than with our engineered mAbs. Thus, Ab engineering to enhance NK cell activation may also improve function by overcoming viral countermeasures. We also investigated the ability of the UL141 mAbs to promote the control of virus using a recently developed 10-day viral dissemination assay (VDA), which captures the effects of both cytotoxic and noncytotoxic virus control in a fully autologous system (Figure 7, D and E, and refs. 54, 55). The UL141 mAbs demonstrated a striking ability to enhance NK-mediated virus control in this assay, confirming that they can act as powerful effectors for long-term control of virus infection, even at low effector/target (E/T) ratios.

## Discussion

Multiple human anti-HCMV mAbs have been developed that target virus neutralization as their mechanism of action (5, 17, 18, 56–58). Although these mAbs offer advantages over HIG, in that they are defined products with a specific activity, the highly cell-associated nature of clinical HCMV strains and the intrinsically greater resistance to neutralization of cell-to-cell spread in comparison with cell-free entry mean that their ability to prevent intra-host spread may be limited (25, 26). In contrast, Ab-mediated activation of cellular immunity does not suffer from these limitations and has been implicated in the control of multiple different viruses, including West Nile virus, smallpox virus, herpes simplex virus, influenza virus, yellow fever virus, Ebola virus, and Epstein-Barr virus. It also correlates with control of HIV in both vaccination and natural infection (59, 60) and is thought to underlie the efficacy of numerous antitumour Abs in clinical development (61, 62). There is thus considerable interest in exploiting this powerful mechanism of control across multiple pathogens and diseases. However, this requires mapping of the antigens that optimally activate ADCC and production of cloned human mAbs capable of mediating ADCC. Our demonstration that plasma membrane proteomics and functional immunology can be combined to identify novel ADCC targets not only opens up a fuller understanding of natural immunity against HCMV that can now be exploited for therapeutic benefit, but is also applicable to exploiting Ab-mediated activation of cellular immunity in other infectious diseases, and potentially even cancer.

As a virus that persists lifelong, HCMV faces major challenges in avoiding being cleared by the immune response and, as a result, has evolved an exceptionally broad range of techniques to limit immune activation (30, 31). The study of these has revealed details about the underlying functioning of the immune system, but also shows that the virus poses a particular challenge to the development of methods to activate antiviral immunity. It is therefore all the more impressive that our technologies enabled the development of Abs capable of reversing the ability of viral immune evasins to inhibit NK cell activation, even when the HCMV strain expressed the complete repertoire of genes present in a clinical isolate (19, 20, 33). In addition to encoding functioning immune evasins, it seems likely that HCMV has evolved to restrict cell-surface expression of viral proteins in order to minimize ADCC. As a result, the extreme sensitivity of mass spectrometry was required in order to identify viral cell-surface antigens. Nevertheless, although cell-surface antigen levels were extremely low, it is clear that ADCC had evolved to be extraordinarily sensitive, with Ab engineering enabling strong NK activation to occur despite Ab binding being undetectable by flow cytometry, underscoring the potential of our pipeline to produce highly effective Abs. The strict species specificity of CMVs and the fact that our primary targets (UL16 and UL141) are not conserved in mouse or rat CMV, and show only 32% homology in rhesus CMV, preclude efficacy testing of our Abs in animal models. Future work will be required to demonstrate both safety and efficacy in humans.

The choice of cell-surface antigen is likely to be an important parameter that defines the efficacy of mAbs that activate ADCC. Surprisingly, the antigens that we identified as mediating ADCC were not the classical viral structural proteins that ADCC studies have traditionally focused on. Our previous proteomics analysis defined 5 temporal classes of viral gene expression (45), with examples from multiple classes found on the infected cell surface. However, targeting those present 48 hpi offers a number of advantages. ADCC activity with polyclonal IgG from seropositive donors was as high at this time point as it was later in infection, implying that many of the antigens that prime ADCC-mediated control in healthy individuals are present within 48 hours. New virions have not yet formed, increasing the chances that cells will be killed before the virus can spread, and the abundance of the proteins we targeted was among the highest of any viral protein, at any time point. In addition, by focusing on nonstructural proteins, there was limited risk of inadvertently enhancing disease through Ab-dependent enhancement (ADE) of infection (63). Although we prioritized UL16 and UL141, US28 or RL11 may also be useful targets if suitable Abs can be generated, although at present this is not simple. Abs targeting US28 in particular could be important, since US28 is expressed during latency and there is evidence that polyclonal Abs targeting this protein can lead to the destruction of latently infected monocytes via neutrophil-mediated ADCC (64). Finally, our target antigens were chosen on the basis of their ability to activate ADCC with HIG. Some of the other cell-surface proteins that we identified may also mediate ADCC effectively, but if they do not induce high Ab levels during natural infection, they would have remained silent in our functional assays. For these proteins, murine immunization strategies could be used to generate additional ADCC-capable mAbs. Likewise, it is possible that some potential targets were missed by our mass spectrometry strategy if the peptides they generated ionized poorly.

It is notable that all of the targets identified in the present study are immune evasion genes. Among its many roles, US28 acts as a cytokine sink on the cell surface (65). UL141 reduces the cell-surface expression levels of CD112 and CD155 (66, 67), which are ligands for the activating NK cell receptor DNAM1, as well as TRAIL receptors (68), while UL16 reduces cell-surface levels of ULBP1–3 and MICB, which bind to the activating NK cell receptor NKG2D (69, 70). It may be that both UL141 and UL16 traffic to the cell surface to scavenge their targets. Accordingly, if viral mutants arose in vivo to evade Ab recognition, infected cells might become more susceptible to NK cell–mediated immune control, which in turn would hinder the widespread selection of such mutants. The use of multiple Abs targeting the same antigen could also limit the selection of viral escape mutations. The sequences of both UL141 and UL16 are well conserved among clinical HCMV isolates, suggesting that Abs targeting them could control a broad range of virus strains (71, 72).

Cloned mAbs offer major advantages over polyclonal products such as HIG. They are defined products with consistent specificity over time, and molecular engineering can be used to optimize functionality for specific purposes. As a result, our mAbs activated ADCC at concentrations over 40-fold lower than that of Cytotect, something that may significantly enhance effectiveness in vivo (73). Furthermore, the generation of anticancer immunotherapies has resulted in the development of multiple different Ab optimizations, which now become amenable to deployment against HCMV. This includes “arming” Abs with drugs or toxins, or converting them into bispecific or trispecific NK engagers to enhance ADCC efficacy even further (74). In addition to ADCC, surface-bound Abs can also activate phagocytosis, complement, and T cells (75) and can lead to an adaptive cellular response by binding to FcRs on DCs. The induction of such mechanisms, in addition to ADCC, has been shown to be effective at mediating tumour control (61, 62), and modifications exist to further optimize these activities (76). Thus, the development of our mAbs provides a platform with which multiple aspects of the immune system can be armed, increasing efficacy in vivo even further. As well as opening up the possibility of exploiting optimized Abs for passive infusion, the cell-surface targets that we have identified could also be considered as part of a vaccine strategy. For example, by vaccinating with UL141 protein, it may be possible to generate a polyclonal anti-UL141 Ab response, which could provide enhanced immunity via Fc-mediated effector functions. In this context, it will be important to determine the efficacy of ADCC Abs in controlling HCMV infection in individuals exhibiting different repertoires of NK cell subsets, including in those who are HCMV seropositive or seronegative and in individuals with larger or smaller numbers of adaptive NK cells.

In conclusion, we have developed a methodological pipeline combining proteomics with functional immunology, single-cell cloning, and molecular engineering that identified novel therapeutic targets; revealed that “classical” cell-surface antigens were not necessarily the optimal targets; avoided potential issues with ADE; and produced Abs capable of binding targets and activating cellular immunity, despite the presence of multiple immune evasins and despite the fact that target expression levels can be too low to detect by flow cytometry. We anticipate that our approach will be generically applicable to other pathogens and tumors, both in terms of passive immunization and vaccine design, with broad implications for immunotherapeutic strategies beyond HCMV. However, here we used it to demonstrate that ADCC is an extraordinarily potent effector mechanism for activating NK cells against HCMV-infected cells. We have identified multiple cell-surface targets for the development of novel antiviral immunotherapies or vaccination strategies that can activate ADCC, and we have generated what we believe to be the first human Abs targeting a single HCMV antigen that are sufficient to activate ADCC. Together, we believe these results open the path for the development of novel immunotherapeutic strategies that can activate multiple different arms of cellular immunity and enable enhanced control of HCMV in vivo.

## Methods

### Cells.

Human fetal foreskin fibroblasts (HFFFs), HFFFs immortalized with human telomerase reverse transcriptase (HF-TERTs) (77), HF-TERTs transfected with the coxsackie adenovirus receptor (HFFF-hCARs) (78), TERT-immortalized healthy donor skin fibroblasts (SFs), and 293 TREX cells (Thermo Fisher Scientific) were grown under standard conditions in DMEM (Thermo Fisher Scientific) supplemented with 10% FCS, penicillin (100 U/m), and streptomycin (100 μg/mL). Expi293F suspension cells (Thermo Fisher Scientific) were maintained in a humidified, shaking incubator at 150 rpm, 37°C, and 8% CO_2_ and were grown in Gibco Expi293 Expression Medium (Thermo Fisher Scientific). Ms40L low cells were a gift from Garnett Kelsoe (Duke University, Durham, North Carolina, USA) and David Baltimore (Caltech, Pasadena, California, USA) (79, 80). They were kept in DMEM supplemented as above with the addition of 50 μM β-mercaptoethanol.

### Viruses.

All viruses were derived from a bacterial artificial chromosome (BAC) containing the complete wild-type HCMV genome, with the exception of RL13 and UL128, since the absence of these genes enhances stability in fibroblasts (20, 81). Mutations were engineered using either recombineering or en passant mutagenesis, as described previously (20, 82–85). The primer sequences are listed in Table 1. Viruses were generated by transfection of BACs (20) into HF-TERTs and titrated on HFFFs. All modifications were sequence verified prior to BAC transfection, and all viruses were sequenced at the whole-genome level following reconstitution to exclude the occurrence of second-site mutations (86).

RAds were generated as described previously (84). They were as follows: RAd-Ctrl (no exogenous protein-coding region); RAd-UL141ΔER (expressing UL141 carrying a deletion of the cytoplasmic tail and an exogenous signal peptide containing an HA tag after the cleavage site); RAd-UL16ΔER (expressing UL16 carrying a deletion of the cytoplasmic tail and an exogenous signal peptide containing an HA tag after the cleavage site); RAd-sUL141 (expressing the UL141 extracellular domain with a C-terminal Strep-tag); RAd-sUL16 (expressing the UL16 extracellular domain with a C-terminal 6xHis-tag); RAd-UL141 (expressing the native form of UL141; ref. 67); and RAd-UL16 (expressing the native form of UL16). RAds expressing other HCMV proteins have been described previously (84), and all contained a C-terminal V5 epitope tag. All RAds were propagated by transfection of the relevant plasmids into 293 TREX cells as described previously (84).

### Proteomics.

Data originally published by Weekes et al. (45) were reanalyzed to estimate the absolute abundance of each cell-surface viral protein. To be included in this analysis, proteins required quantitation, in both experiments PM1 and PM2, of 2 or more peptides in at least 1 of the 2 experiments. Overall, this included 27 of 29 of the viral proteins we originally measured. Experiment PM1 examined cells infected with strain Merlin in biological duplicates at 0 hours, 24 hours, 48 hours, and 72 hours. Reanalysis was based on the mean values for each time point. Experiment PM2 examined cells infected with the same HCMV strain in single replicates at 0 hours, 6 hours, 12 hours, 18 hours, 24 hours, 48 hours, 72 hours, and 96 hours. In reanalysis, the mean values for time point 0 were used, and infection with irradiated HCMV at 12 hours was excluded from analysis. In Figure 2A, for experiment PM2 data, the proteins were grouped according to when greater than 25% of the maximum signal was reached. Abundance for each protein was normalized to a maximum of 1, as described previously (45). For Figure 2B, the method of intensity-based absolute quantification (IBAQ) was adapted from the original description (87) to estimate the relative abundance of each of the 27 viral proteins. The maximum MS1 precursor intensity for each quantified peptide was determined, and a summed MS1 precursor intensity for each protein across all matching peptides was calculated, considering data for experiments PM1 and PM2 separately. Intensities were divided by the number of theoretical tryptic peptides from each protein between 7 and 30 amino acid residues in length to give estimated IBAQ values. For each of experiments PM1 and PM2, the estimated IBAQ values were divided by the sum of all values to give the normalized IBAQ values. The average and range of the normalized IBAQ values for each protein are shown in Figure 2, B and C. To determine the proportion of the average normalized IBAQ values that arose at each time point of infection, the IBAQ values were adjusted in proportion to the normalized tandem mass tag (TMT) values shown in Figure 2A.

### Protein purification and labeling.

Soluble UL141 and UL16 were produced in HFFF-hCARs transduced with RAd-sUL141 or RAd-sUL16, respectively, over a 10-day period at a MOI of 40 PFU/cell. Supernatants were collected and purified using Strep-Tactin (IBA GmbH) or HisTrap HP Columns (GE Healthcare). Both proteins were subjected to buffer exchange in PBS and fluorescently labeled using the Alexa Fluor 647 Protein Labeling Kit (Thermo Fisher Scientific).

### Ab isolation.

PBMCs were isolated from a healthy HCMV-seropositive donor, and IgG^+^ memory B cells were isolated using an IgG^+^ Memory B Cell Isolation Kit (Miltenyi Biotec). The enriched B cells were stained for 30 minutes at 4^o^C with 2 μg/mL Alexa Fluor 647–labeled protein (soluble UL141 or UL16) and flow sorted using a BD FACSAria III (BD Biosciences). Single cells were sorted into individual wells containing Ms40L low feeder cells, 10% FCS, 5% human AB serum, IL-4 (10 ng/mL), BAFF (10 ng/mL), IL-21 (10 ng/mL), and IL-2 (50 ng/mL) in a final volume of 100 μL (all cytokines were from Peprotech). Cultures were supplemented with an additional 100 μL of the same medium 1 week later. Two weeks after coculturing, 50 μL supernatant from each of the single-cell colonies was screened by flow cytometry for binding to UL141 (RAd-UL141ΔER) and UL16 (RAd-UL16ΔER). RNA was extracted from the cells that were positive for binding using the RNEasy Plus Kit (QIAGEN). The Ab sequence was determined by nested reverse transcription PCR (RT-PCR) as described previously (88). Sequences were analyzed by the IgBLAST tool to identify the V and J composition of the heavy and light chains, then PCR amplified using specific primers and cloned separately into an expression plasmid containing a human IgG1 constant domain, provided by Patrick Wilson (University of Chicago, Chicago, Illinois, USA) (88).

### Ab engineering.

S239D and I332E modifications were introduced into the Fc region of each mAb by Gibson assembly (52). The 2 fragments of the plasmid, containing overlapping regions with the desired modifications, were generated using the following primer sequences: 5′-GGGGGACCGGACGTCTTCCTCTTCCCCCCA-3′ and 5′-GGTTTTCTCCTCGGGGGCTGGGAGGG-3′, or 5′-AGGAAGACGTCCGGTCCCCCCAGGAG-3′ and 5′-CAGCCCCCGAGGAGAAAACCATCTCCAAAGCCA-3′. The resulting fragments were assembled using the NEBuilder HiFi DNA Assembly Cloning Kit (New England Biolabs).

### Ab production and purification.

Expi293F suspension cells were pelleted, resuspended at 20 × 10^6^ cells/mL, and transfected with the relevant light and heavy chain plasmids at a ratio of 70:30 (1.25 μg/10^6^ cells of total plasmid DNA) using polyethylenimine (PEI) diluted in ultrapure water (3.75 μg/10^6^ cells) and 0.1% Pluronic F-68 (89). Transfected cells were cultured for 3 hours and subsequently diluted to 10^6^ cells/mL with Expi293 Expression Medium containing forskolin (10 μM). Ab-containing supernatants were collected 7 days after transfection.

Both mAbs and Abs from the serum of seronegative donors were purified as described previously (88). Briefly, supernatants were filtered through a 0.45 μm syringe filter and incubated overnight at 4˚C with protein G agarose beads. The following day, the bead-supernatant reactions were transferred to room temperature for 2 hours and then centrifuged at 3000*g* for 10 minutes. The beads were transferred to a chromatography column, washed with 5 resin-bed volumes of 1 M NaCl, and eluted twice with 2.5 resin-bed volumes of PBS. Abs were eluted into Tris-HCl, pH 9.0, with 2.5 resin-bed volumes of glycine buffer, pH 2.8 (Pierce, Thermo Fisher Scientific), ensuring that the final pH was approximately 7.0. The Abs were subsequently subjected to buffer exchange against PBS.

### CD107a Assays.

Degranulation assays were based on the flow cytometric detection of CD107a (90). PBMCs were rested overnight in RPMI supplemented with 10% FCS, penicillin (100 U/mL), streptomycin (100 μg/mL), and l-glutamine (2 mM) in the absence or presence of IFN-α (1000 U/mL). HF-TERTs (allogeneic) or SFs (autologous) were plated in DMEM without FCS and infected the following day with HCMV (MOI = 5 PFU/cell). The medium was replaced 24 hpi with DMEM containing 10% FCS. Assays were performed 48 hpi unless stated otherwise. Targets were harvested using TrypLE Express (Gibco, Thermo Fisher Scientific), preincubated for 30 minutes with the relevant Ab preparations, and mixed with PBMCs at an E/T ratio of 10:1 in the presence of GolgiStop (0.7 μL/mL, eBioscience) and anti–CD107a–PerCP-Cy5.5 (clone H4A3, BioLegend). Assays were performed in triplicate in U-bottomed, 96-well plates at a final volume of 200 μL/well. Background activation was determined in wells containing effectors without targets. Cells were incubated for 5 hours, washed in cold PBS, and stained with LIVE/DEAD Fixable Aqua (Thermo Fisher Scientific), anti–CD3-BV711 (clone UCHT1, BioLegend), anti–CD56-BV605 (clone 5.1H11, BioLegend), anti–CD57-APC (clone HNK-1, BioLegend), and anti–NKG2C-PE (clone 134591, R&D Systems). In some experiments, cells were also fixed and permeabilized using Cytofix/Cytoperm (BD Biosciences) and stained with anti–TNF-α–BV421 (clone MAb11, BioLegend) and anti–IFN-γ–PE–Cy7 (clone B27, BioLegend). Data were acquired using an Attune NxT Flow Cytometer (Thermo Fisher Scientific) and analyzed with Attune NxT software or FlowJo software, version 10 (Tree Star). All assays were repeated with samples from multiple donors. When used directly ex vivo, NK cells from different donors can vary significantly in the magnitude of their responses, thus, only experiments where results showed consistent patterns between donors are included. Donors included both HCMV-seropositive and -seronegative individuals.

### Chromium release cytotoxicity assays.

Assays were performed as previously described (91). In brief, targets were incubated with 150 μCi sodium chromate (^51^Cr) for 1 hour, washed and allowed to leach for 1 hour, and then incubated with purified NK cells and Abs. After 4 hours, supernatants were removed and mixed with scintillation fluid (Optiphase HiSafe 3, PerkinElmer), before reading the cpm in a MicroBeta 2 (PerkinElmer). Maximum lysis was generated using 2.5% Triton X-100. Specific lysis was calculated as follows: (sample cpm – spontaneous cpm)/(maximum cpm – spontaneous cpm).

### Viral dissemination assays.

Assays were performed as previously described (54). Briefly, SFs were infected at a MOI of 0.05 with a virus containing a P2A-mCherry cassette after UL36, and an EGFP tag directly fused to UL32. At 24 hpi, purified ex vivo (NK Isolation Kit, Miltenyi Biotec) autologous NK cells were added at a range of E/T ratios, in the presence or absence of Abs. After 8–10 days, nonadherent cells were washed off and discarded, and adherent cells were trypsinized, fixed in 4% PFA and analyzed by flow cytometry for mCherry and/or EGFP expression. To determine levels of the NK-mediated control, the percentage of fluorescent cells in the presence of Ab and NK cells was normalized to the percentage of fluorescent cells in the presence of Ab alone.

### Immunoblotting.

HFFF-hCARs were transduced with RAd-UL141 or RAd-UL16 (MOI = 5 PFU/cell) for 48 hours. Whole-cell lysates were collected and boiled in reducing-denaturing Nu-PAGE lysis buffer (Thermo Fisher Scientific), separated by electrophoresis in Criterion TGX Gels (Bio-Rad) and transferred onto nitrocellulose membranes (GE Life Sciences). Membranes were blocked in TBS-T buffer with 5% dried nonfat milk and stained with either anti-V5 (clone CV5-Pk1, Bio-Rad) or anti-actin (A2066, MilliporeSigma) Abs. Proteins were visualized with SuperSignal West Pico PLUS Chemiluminescent Substrate (Thermo Fisher Scientific) and imaged on a GBOX-Chemi-XX6 gel documentation system (Syngene) operating GeneSys software.

### Study approval.

Healthy adult donors provided written informed consent for the collection of venous blood samples and dermal fibroblasts according to the principles of the Declaration of Helsinki. Study approval was granted by the Cardiff University School of Medicine Research Ethics Committee (reference number 16/52).

### Statistics.

Statistical significance was determined using a 1- or 2-way ANOVA as appropriate, with Sidak’s post tests. A *P* value of 0.05 or less was considered significant.

## Author contributions

VMV, IM, RJA, KL, DAP, AJD, GWGW, MRW, ECYW, and RJS designed experiments. VMV, IM, LZ, RJA, EL, MRW, KLM, NMS, MPW, and RJS performed experiments and analyzed data. VMV, DAP, AJD, GWGW, MPW, ECYW, and RJS wrote the manuscript.

## Supplementary Material

Supplemental data

## Figures and Tables

**Table 1:**
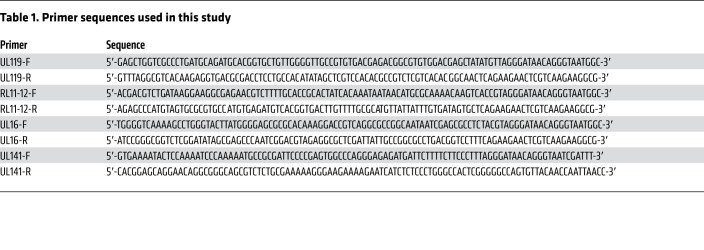
Primer sequences used in this study

## Version 1 02/15/2021

Electronic publication
