# Supplementary material for: Monoclonal antibodies targeting nonstructural viral antigens can activate ADCC against human cytomegalovirus
Source: J Clin Invest. 2021 Feb 15;131(4):e139296. doi: 10.1172/JCI139296 (PMC7880312; doi:10.1172/JCI139296)
Supplement: Supplemental data [file jci-131-139296-s126.pdf]

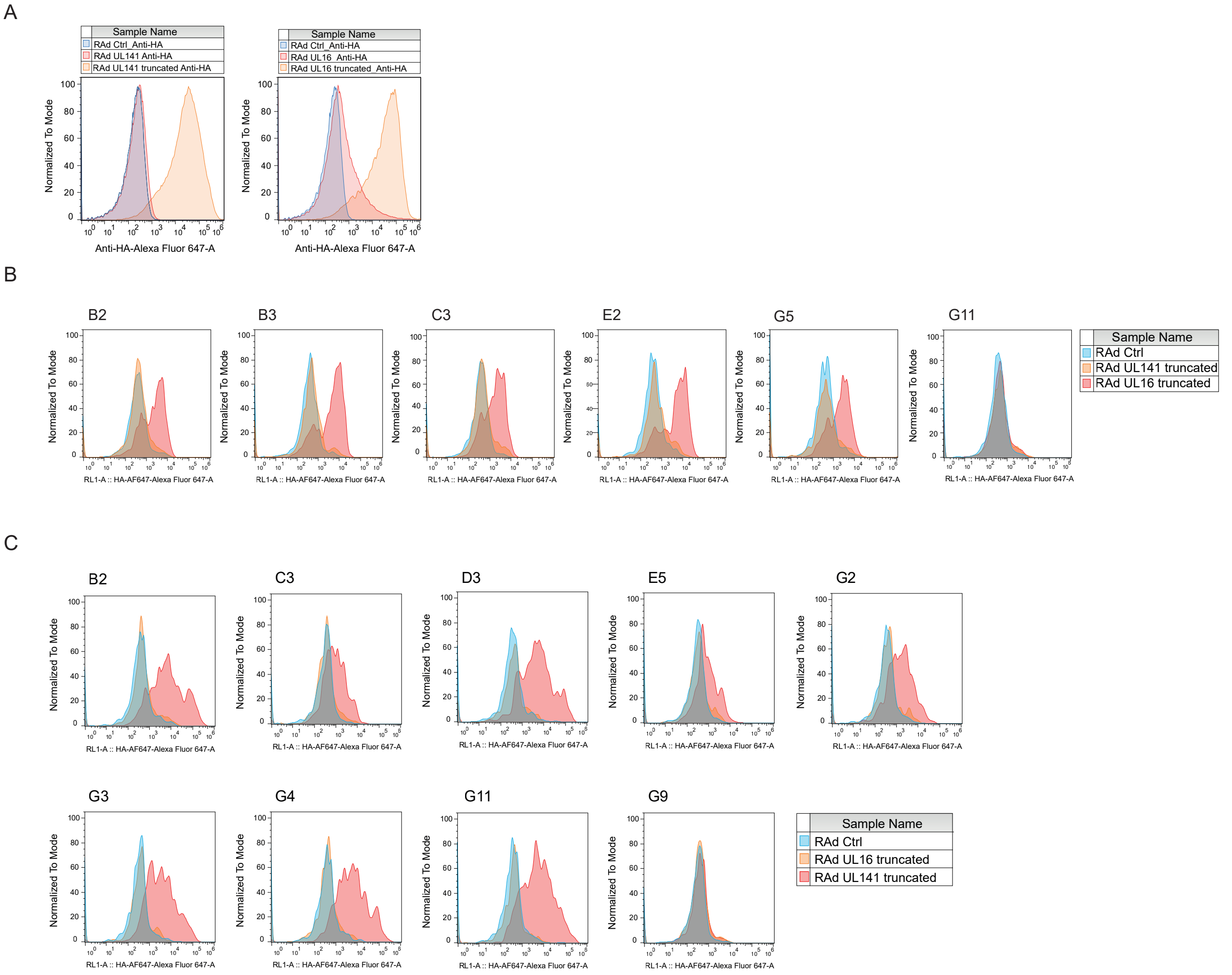

Supplementary Fig.1: Screening of B-cell supernatants against UL16 and UL141 revealed five mAbs that bound UL16 and nine mAbs that bound UL141. (**A**) HFFF-hCARs were transduced with RAds expressing native or ER retention signal-truncated UL16 or UL141. An identical vector lacking a transgene was used as a control. Transduced cells were stained with a mAb specific for the HA-tag engineered into the N-terminus of each protein. **(B, C)** HFFF-hCARs were transduced with RAds expressing ER retention signal-truncated UL16 **(B)** or UL141 **(C)**. An identical vector lacking a transgene was used as a control. Transduced cells were stained with cloned B cell supernatants and an anti-human IgG-AF647 secondary mAb. Positive clone supernatants and a representative negative clone supernatant are shown for each protein (total n = 60). ctrl, control. Data are shown as flow histograms and are representative of at least two experiments **(A–C)**.

A

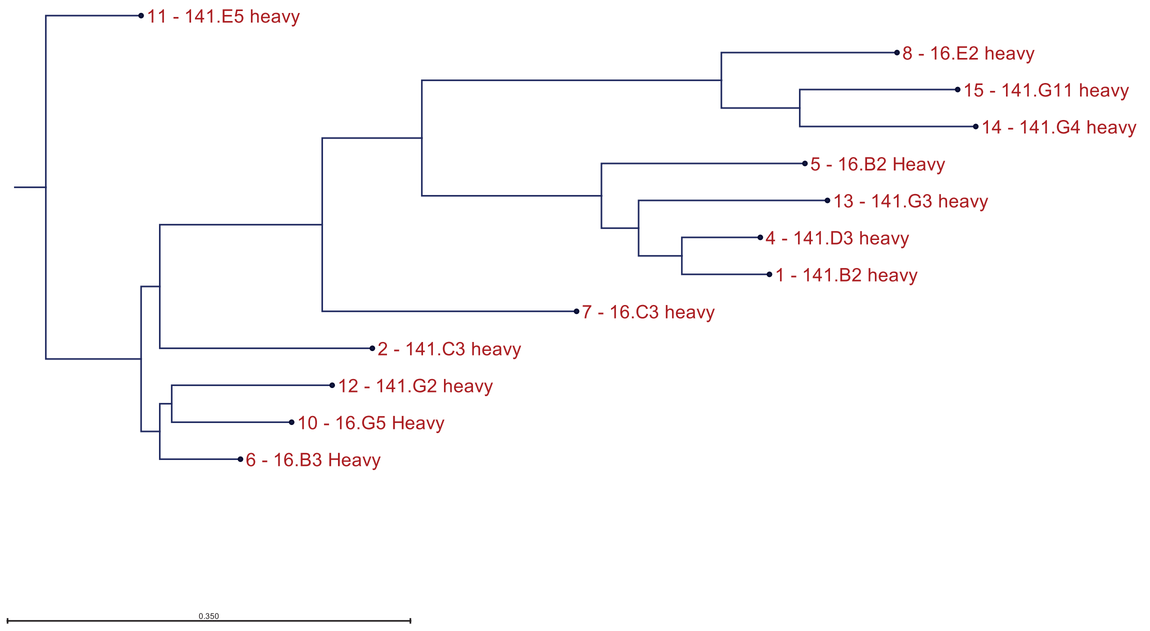

B

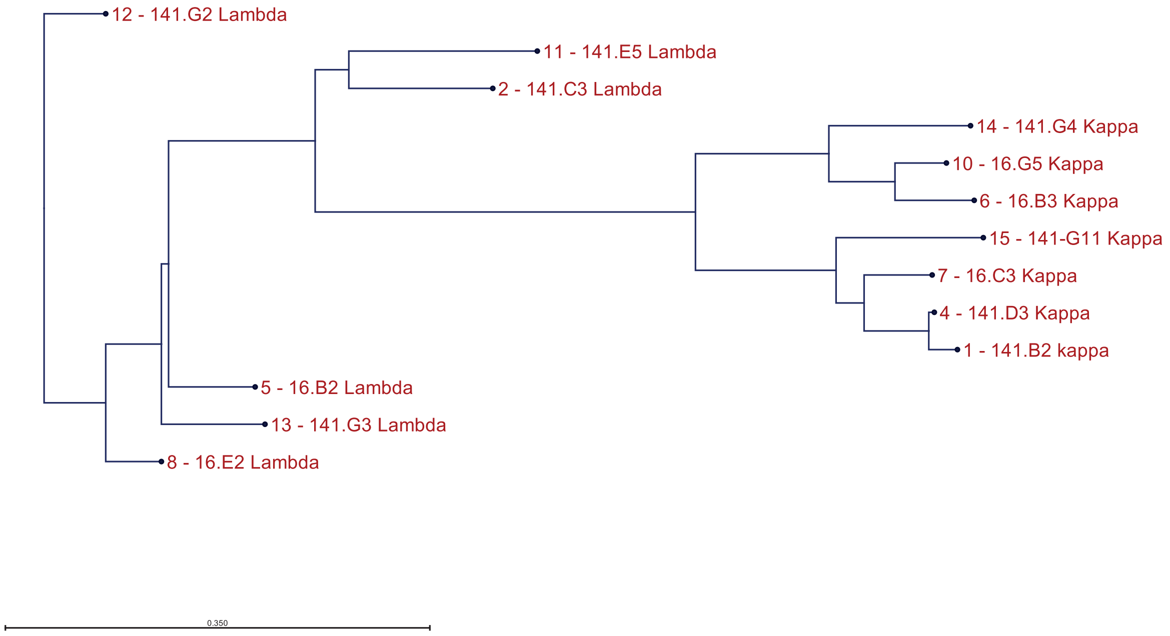

Supplementary Figure 2: The sequences of the heavy **(A)** or light **(B)** chain of the B-cell receptor for each antibody were aligned, then a neighbour joining tree constructed using CLC Main.

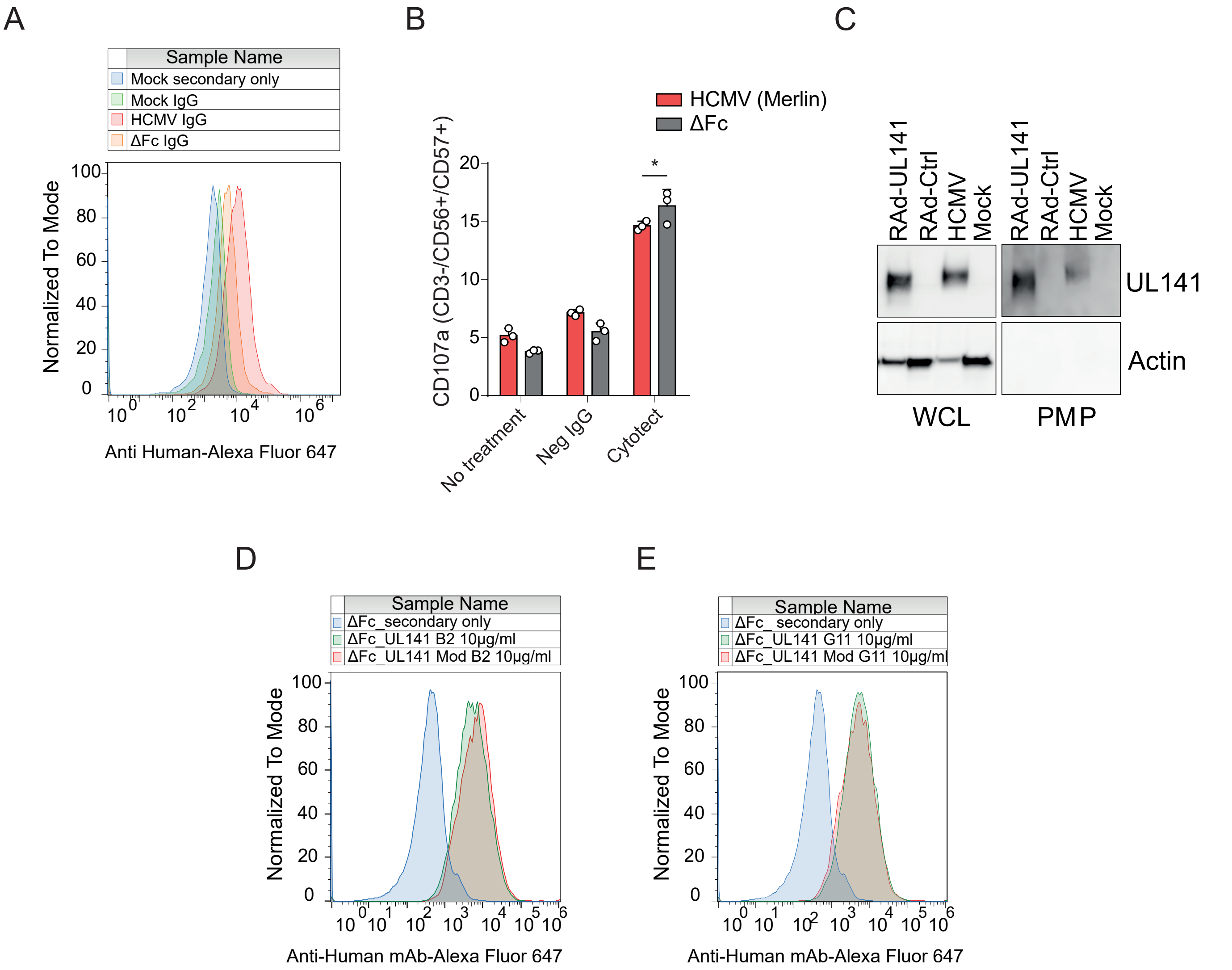

Supplementary Figure 3: Viral Fc receptors do not have a major impact on ADCC activity at 48 hours post infection. HF-TERTs were infected with HCMV strains Merlin or Merlin ΔFc **(A-C)** or Merlin ΔUL141 **(D, E)**. HF-CAR were infected with RAd expressing UL141, or control RAd lacking a transgene **(C)**. **(A)** Infected cells were stained with fluorochrome-labeled Cytotect (100 μg/ml). Data are shown as flow histograms. **(B)** Percent degranulation of CD56+ CD57+ NK cells among PBMCs in the presence of infected cells and either Cytotect or seronegative IgGs (each at 40 μg/ml). Infected cells alone were included as a control. Data are shown as mean ± SD of triplicate samples. **(C)** Plasma membrane proteins (PMP) were oxidised and aminoxy-biotinylated, before being immunoprecipitated with streptavidin beads, and lysed in SDS-PAGE buffer. Whole-cell lysates (WCL) prior to IP were lysed directly in SDS-PAGE buffer. Proteins were separated by SDS-PAGE, western blotted, and stained using anti-UL141 monoclonal antibodies. **(D, E)** Infected cells were stained with the native or Fc-modified forms of the UL141-specific mAbs B2 **(C)** or G11 **(D)**, each at a concentration of 10 μg/ml, and analyzed for binding to viral FcRs. Data are shown as flow histograms. All experiments were performed 48 h after infection **(A–D)**. *$p < 0.05$ (two-way ANOVA).
